# AI anxiety and AI dependence among undergraduates: a moderated mediation model of AI self-efficacy and AI literacy

**DOI:** 10.3389/fpsyg.2026.1884382

**Published:** 2026-07-13

**Authors:** Houyu Wu, Haiyang Ni, Jiaqi He, Eng Hoe Wee

**Affiliations:** 1 Department of Personnel, Neijiang Normal University, Neijiang, Sichuan, China; 2 Faculty of Education, Languages, Psychology and Music, SEGi University, Petaling Jaya, Malaysia; 3 Graduate School of Business, Nilai University, Nilai, Malaysia

**Keywords:** AI anxiety, AI dependence, AI literacy, AI self-efficacy, cognitive behavioral theory, conservation of resources theory, undergraduates

## Abstract

**Background:**

The rapid integration of Artificial Intelligence (AI) in higher education offers transformative learning potential but has concurrently triggered psychological challenges, specifically AI anxiety and AI dependence. While these phenomena are increasingly prevalent, the psychological mechanisms converting affective strain into behavioral reliance remain underexplored. Grounded in Cognitive Behavioral Theory and Conservation of Resources Theory, this study investigates the relationship between AI anxiety and AI dependence among undergraduates. Specifically, it examines whether AI self-efficacy mediates this relationship and whether AI literacy acts as a boundary condition moderating these pathways.

**Methods:**

A cross-sectional survey was conducted with 400 undergraduates recruited via stratified sampling from a normal university in Southwest China. Participants completed validated scales measuring AI anxiety, AI self-efficacy, AI literacy, and AI dependence. Confirmatory factor analysis was performed to verify instrument validity. Hypotheses were tested using the PROCESS macro (Models 4 and 8) to analyze mediation and moderated mediation effects, utilizing bootstrapping techniques with 5,000 resamples to determine the statistical significance of direct and indirect effects.

**Results:**

The analysis revealed a significant positive association between AI anxiety and AI dependence. AI self-efficacy was found to partially mediate this relationship, indicating that anxiety exacerbates dependence by eroding students’ confidence in their capabilities. Furthermore, AI literacy served as a significant moderator. Results indicated that the negative impact of AI anxiety on AI self-efficacy was stronger for students with higher AI literacy, suggesting that high literacy may sensitize students to competency gaps under emotional strain. AI literacy did not moderate the direct relationship between anxiety and dependence.

**Conclusion:**

This study identifies AI dependence as a maladaptive coping response to AI anxiety, driven by diminished AI self-efficacy. The findings challenge the assumption that literacy acts solely as a protective buffer, revealing that without emotional regulation, high literacy may intensify the erosion of self- confidence during anxious states. To mitigate maladaptive dependence, higher education institutions should move beyond technical training to adopt holistic strategies. These should include psychological support to build resilience, mastery experiences to foster self-efficacy, and comprehensive curricula that address the emotional and cognitive dimensions of human-AI interaction.

## 1 Introduction

The integration of Artificial Intelligence (AI) into higher education has evolved from a novelty to a fundamental component of the academic ecosystem, offering transformative potential for undergraduates’ learning (Baig and Yadegaridehkordi, 2024). Empirical evidence demonstrates that when utilized effectively, AI systems act as powerful catalysts for academic productivity rather than mere shortcuts (Deng et al., 2025; Theoharakis et al., 2025). For instance, a study by Wang J. et al. (2024) revealed that undergraduates utilizing AI tools for writing tasks reduced time expenditure by approximately 21% while simultaneously elevating the quality of their output. Furthermore, global surveys indicate that over 86% of university students now integrate AI into their weekly study routines to explain complex concepts, summarize literature, and generate research ideas (Zaidy, 2024). These findings underscore that AI has successfully lowered the barrier to knowledge acquisition, enabling students to engage in personalized learning pathways and interdisciplinary inquiry with a level of efficiency previously unattainable.

However, this rapid technological adoption has triggered a paradoxical “double-edged sword” effect, fostering an intricate interplay of psychological and behavioral challenges for undergraduates (Ivanov et al., 2024). Recent educational reports highlight that the opacity of AI algorithms and the speed of their evolution have triggered widespread AI Anxiety, characterized by a deep-seated fear of academic devaluation and future professional displacement (Wang Y. M. et al., 2024). Statistics from the Cengage Group (2024) employability survey indicate that nearly 70% of university students believe AI training is urgent to avoid obsolescence, while significant portions (39%) explicitly fear that AI will render their future entry-level jobs obsolete. This intense anxiety often coexists with maladaptive usage patterns known as AI dependence (Zhang et al., 2024). Empirical studies on “cognitive offloading” suggest that a significant portion of students are habitually deferring critical thinking processes to algorithms, engaging in unverified reliance on AI recommendations (Klingbeil et al., 2024; Zhai et al., 2024). Consequently, a synthesis of the current empirical landscape reveals that university students are currently situated in a precarious psychological state: they are heavily dependent on AI tools to fulfill academic demands while simultaneously suffering from the anxiety that these tools serve as a threat to their professional future.

To address and mitigate these emerging challenges, existing literature has extensively explored the roles of individual cognitive and capability factors, specifically AI self-efficacy and AI literacy (Schiavo et al., 2024). Empirical studies indicate that students with stronger AI self-efficacy tend to report lower AI anxiety (Türk et al., 2025), and stronger efficacy-beliefs are closely linked to lower technostress exposure and better psychological adjustment in digital learning environments (Zhang, 2023). On the behavioral side, findings further suggest that undergraduates with higher self-efficacy are less likely to develop passive reliance patterns and display lower tendencies toward problematic or dependent AI use (Zhang et al., 2024). Meanwhile, AI literacy has been identified as a critical coping resource for AI anxiety and dependence. Empirical evidence demonstrates that higher AI literacy can effectively alleviate technology-induced anxiety by demystifying the “black box” of AI (Cengiz and Peker, 2025), while students lacking sufficient AI literacy are more prone to developing blind dependence of AI tools (Liu, 2026).

Nevertheless, a critical research gap persists. Although the relationships between these variables have been examined individually, the intrinsic link between AI Anxiety and AI Dependence remains insufficiently explored. Specifically, the mechanism by which anxiety translates into behavioral dependence has not been fully articulated. Given that AI tools are evolving rapidly and remain difficult for students to appraise, recent scholarship underscores the urgent need to integrate affective and behavioral consequences within a unified explanatory model, rather than treating anxiety and reliance as separate strands (Liu and Zhong, 2025). Furthermore, while AI self-efficacy and AI literacy are widely recognized as protective resources, their specific roles in this dynamic are unclear. It remains to be determined whether self-efficacy operates as the psychological pathway bridging anxiety and dependence, and whether AI literacy functions as a boundary condition that buffers these relationships. Accordingly, this study addresses these identified gaps by proposing the following research questions:

1. Is AI Anxiety significantly associated with university students’ AI Dependence?

2. Does AI Self-Efficacy mediate the relationship between AI Anxiety and AI Dependence?

3. Does AI Literacy moderate the relationships among AI Anxiety, AI Self-Efficacy, and AI Dependence?

## 2 Literature review and hypothesis development

### 2.1 Theoretical foundation

#### 2.1.1 Cognitive behavioral theory

Cognitive Behavioral Theory (CBT), originating from the foundational work of Beck (1979), posits that individual behavior is not an isolated occurrence but is fundamentally shaped by the linear interplay between emotional states and cognitive processes. Although rooted in clinical psychology, CBT has transitioned into a robust explanatory framework within educational psychology and information systems research. Scholars in these fields utilize this theory to analyze how users adapt to novel technologies by examining the psychological chain reaction from affect to cognition and finally to behavior (Chen et al., 2021; Wu-Ouyang, 2022). In the context of higher education, Compeau and Higgins (1995) utilized a CBT-aligned framework to demonstrate that the emotional affect of students significantly distorts their cognitive judgments regarding their own abilities. They found that high levels of computer anxiety negatively influence self-efficacy and thereby inhibit effective technology usage. This perspective suggests that emotional distress consumes cognitive resources and leads to avoidance behaviors or ineffective utilization of tools.

More recently, studies on technostress in higher education have further validated this theoretical lens. Research indicates that negative emotional reactions to digital learning environments lead directly to maladaptive behavioral outcomes. For instance, Wang et al. (2020) observed that students facing technological pressure often exhibit reduced academic engagement or compulsive usage patterns. These findings mirror the core tenets of CBT by illustrating that environmental stressors trigger an emotional response that subsequently alters cognitive appraisal and dictates behavioral responses. In addition, based on CBT, existing work on AI dependence in higher education explicitly positions AI dependence as the downstream behavioral outcome of students’ affective and cognitive shifts, defining it as a persistent pattern of excessive reliance on AI tools for academic purposes (Wu et al., 2026). Consequently, in this study, CBT serves as the overarching logic to elucidate the mediation mechanism regarding Artificial Intelligence. It explains how AI anxiety functions as the emotional antecedent that triggers the depletion of AI self-efficacy. This erosion of cognitive confidence ultimately manifests as AI dependence.

#### 2.1.2 Conservation of resources theory

The Conservation of Resources (COR) Theory, originating from the foundational work of Hobfoll (1989), suggests that individuals are fundamentally motivated to protect their current resources and acquire new ones. This theory argued that psychological stress does not occur in a vacuum but arises specifically when these valued resources are threatened with loss, or when an investment of resources fails to yield the expected return. When individuals possess a robust reservoir of resources, they are less vulnerable to stress and better positioned to prevent further resource depletion. Conversely, a lack of resources can lead to a loss spiral where initial stress exacerbates the depletion of remaining assets.

This theory has been extensively applied in higher education to understand student burnout and coping mechanisms. For instance, Liu and Wang (2026) empirically demonstrated that university students experiencing academic burnout and related distress are less likely to deteriorate further when they possess sufficient personal resources, indicating that resource availability can buffer the impact of academic demands. In the realm of educational technology, scholars view technical competence as a vital resource that enables students to frame technological changes as manageable challenges rather than existential threats (Pan and Zhu, 2025), and research further shows that users’ knowledge and capability appraisals shape how they emotionally and cognitively respond to AI-related change and uncertainty (Yan and Zhang, 2025). Furthermore, COR-based evidence indicates that AI-related knowledge resources influence whether AI is appraised as a challenge or a threat and thereby alter downstream adjustment outcomes (He et al., 2024). In parallel, higher education studies consistently show that stronger AI literacy is associated with lower AI-related anxiety and fear responses (Cengiz and Peker, 2025; Polat, 2025), and generative AI literacy is similarly linked to reduced AI competence anxiety through more adaptive efficacy-related beliefs (Kaya et al., 2026). Thus, COR theory underpins the boundary conditions of the proposed model in this study. Based on COR theory, in this study AI literacy functions as a critical personal resource that buffers the detrimental propagation of anxiety. By equipping students with the knowledge to understand and control AI tools, literacy prevents the emotional experience of anxiety from eroding self-efficacy or dependence on AI use.

Taken together, CBT and COR theory provide complementary rather than separate explanations for the present model. CBT explains the internal psychological sequence through which AI anxiety, as an affective state, weakens students’ AI self-efficacy and subsequently increases AI dependence as a maladaptive behavioral response. COR theory further specifies why this sequence may vary across students by emphasizing the role of AI literacy as a personal resource that shapes how anxiety is appraised and managed. Within this integrated framework, AI self-efficacy represents the cognitive appraisal mechanism that translates affective strain into reliance behavior, whereas AI literacy represents the resource condition that changes the strength of this appraisal process. Therefore, the core objective of this study is not only to examine whether AI anxiety predicts AI dependence, but also to clarify how competence beliefs and literacy resources jointly explain when and why anxiety becomes behavioral dependence in undergraduate AI use.

### 2.2 Hypothesis development

#### 2.2.1 AI anxiety and AI dependence

Grounded in the CBT framework, AI anxiety is defined as the emotional antecedent characterized by apprehension regarding the potential threats of AI systems. This construct captures the uneasiness and fear students experience concerning the opacity of algorithms and the rapid evolution of technology (Wang and Wang, 2022). AI dependence refers to the behavioral outcome of habitual reliance on these systems. It represents a state in which students surrender their cognitive autonomy and become unable to complete academic tasks without algorithmic assistance (Wu et al., 2026).

The assertion that anxiety drives dependency is well supported by a hierarchy of empirical evidence ranging from general psychology to specific educational technology contexts. In general psychology, research on anxiety consistently indicates that individuals experiencing high anxiety tend to engage in safety-seeking behaviors to reduce distress and regain a sense of control (Rapee et al., 2023). From the perspective of CBT, this behavioral pattern represents a maladaptive coping strategy where the individual seeks to reduce emotional discomfort through external reliance rather than internal regulation. This pattern is mirrored in the context of higher education technology usage. For example, studies among university students have confirmed that anxiety-related concerns, such as the fear of missing out, are closely linked to problematic and dependent patterns of digital technology use (Li et al., 2022). This suggests that when digital tools offer immediate relief from psychological strain, students are compelled to form dependent relationships with them to maintain emotional stability.

Translating this to the current era of Generative AI, the relationship appears even more pronounced due to the unique capabilities of these tools. Recent empirical findings suggest that anxiety-related drivers can meaningfully increase problematic reliance on conversational AI systems, reinforcing unhelpful dependence tendencies rather than balanced use (Hu et al., 2023). Related evidence on ChatGPT use further indicates that problematic patterns of use are closely intertwined with heightened anxiety experiences, consistent with the view that anxiety can be embedded in the formation and maintenance of compulsive reliance on these tools (Duong et al., 2024). Consequently, AI shifts from being an assistant to becoming a necessity for task completion.

Collectively, higher AI anxiety triggers a psychological need for risk reduction that manifests as behavioral dependence. When students perceive AI as a threat but feel compelled to use it, they resort to uncritical reliance to bypass the anxiety associated with independent performance. Accordingly, this study proposes the following hypothesis.

*H1*: AI anxiety is positively associated with university students’ AI dependence.

#### 2.2.2 The mediating role of AI self-efficacy

To explicate the pathway from anxiety to dependence, this study introduces AI self-efficacy as a mediating cognitive resource. Drawing from CBT, this construct is defined as a student’s belief in their capability to utilize AI tools effectively to achieve academic goals. Existing literature consistently affirms that anxiety acts as a drain on cognitive resources and thereby diminishes self-efficacy (Ma, 2025). In the context of higher education, empirical research has established that students experiencing technology-related anxiety and stress in technology-enhanced learning consistently report maladaptive psychological outcomes such as burnout and reduced perceived performance, reflecting the depletion of resources required for effective engagement (Wang et al., 2020). This negative pattern extends to the specific context of AI. Evidence indicates that students’ AI-related anxiety is closely intertwined with their AI self-efficacy in learning and using AI tools, such that anxiety is linked to weakened confidence in managing AI-enabled tasks (Chen et al., 2024). Consequently, anxiety undermines the cognitive appraisal of competence.

Furthermore, cognitive self-appraisal directly dictates behavioral outcomes, which is defined as AI dependence in the current study based on CBT. In the field of digital learning, students who experience higher levels of technostress show diminished academic functioning and are more likely to exhibit maladaptive responses to technology demands rather than engage in effective self-regulation (Upadhyaya and Vrinda, 2021). In the context of generative AI, this manifests as cognitive offloading. Recent evidence shows that AI dependence is associated with reduced critical thinking engagement, indicating that students may surrender cognitive autonomy and rely on AI outputs rather than sustaining independent reasoning (Tian and Zhang, 2025). Conversely, stronger AI self-efficacy is associated with more adaptive patterns of AI learning and use, supporting more agentic engagement rather than uncritical reliance (Chen et al., 2024). Synthesizing these findings through the CBT lens, a clear mediation mechanism emerges. AI anxiety first erodes the cognitive resource of self-efficacy, and this diminished self-belief subsequently necessitates a compensatory behavioral response characterized as dependence. Based on this framework, the following hypotheses are proposed.

*H2*: AI anxiety is negatively associated with AI self-efficacy.

*H3*: AI self-efficacy is negatively associated with AI dependence.

*H4*: AI self-efficacy mediates the relationship between AI anxiety and AI dependence.

#### 2.2.3 The moderating role of AI literacy

While anxiety exerts pressure on students, the COR theory suggests that individuals are motivated to protect their current resources and acquire new ones to withstand stress. According to this framework, psychological stress arises specifically when valued resources are threatened or lost (Farkash et al., 2022). However, the possession of key personal resources can buffer this stress and prevent the depletion of other assets (Schiff et al., 2025). In this study, AI literacy is identified as a critical protective resource based on COR theory. It encompasses a comprehensive understanding of the capabilities, limitations, and ethical implications of AI systems (Wang et al., 2023). When students face the emotional strain of AI anxiety, those equipped with high AI literacy possess a resource reservoir that allows them to navigate technological challenges without resorting to maladaptive coping mechanisms (Bećirović et al., 2025).

From this perspective, AI literacy may moderate the relationship between AI anxiety and AI dependence. Students with high AI literacy possess a clearer understanding of the fallibility of technology and are better equipped to engage in critical evaluation. Research on digital competence in higher education highlights that technological demystification significantly reduces maladaptive behaviors. For instance, Hou et al. (2025) found that when students report high levels of AI literacy, they are able to critically evaluate technological outputs rather than accepting them passively. Consequently, even when these literate students experience anxiety about the future, their knowledge acts as a buffer that prevents them from resorting to blind dependence. They are able to distinguish between using AI for assistance versus relying on it for substitution. In contrast, for students with low AI literacy, anxiety is more likely to translate directly into dependence as they lack the cognitive resources to formulate alternative coping strategies (Liu, 2026; Tian and Zhang, 2025). Under high AI anxiety, these students are more likely to surrender control to the system as a means of immediate relief.

Meanwhile, AI literacy may also protect the cognitive resource of self-efficacy against the erosive effects of anxiety. Chen et al. (2024) examined the role of digital literacy in mitigating technostress among university students and found that literacy provides a sense of control over the digital environment. For high-literacy students, AI anxiety is framed as a manageable challenge regarding the tool rather than a threat to their competence (Bećirović et al., 2025). Their foundational knowledge mitigates the damaging effect of anxiety on their self-confidence because they do not interpret their emotional arousal as a sign of total incompetence. Conversely, low-literacy students lack this protective resource and are more susceptible to the erosion of self-efficacy when facing AI-induced anxiety. When these students feel anxious, they are more likely to internalize this emotion as a lack of capability, which accelerates the loss of self-efficacy (Chen et al., 2024). Therefore, AI literacy functions as a boundary condition that determines the extent to which anxiety damages self-belief. Based on the above analysis, this study proposes the following hypothesis.

*H5*: AI literacy moderates the relationship between AI anxiety and AI dependence.

*H6*: AI literacy moderates the relationship between AI anxiety and AI self-efficacy.

**FIGURE 1:**
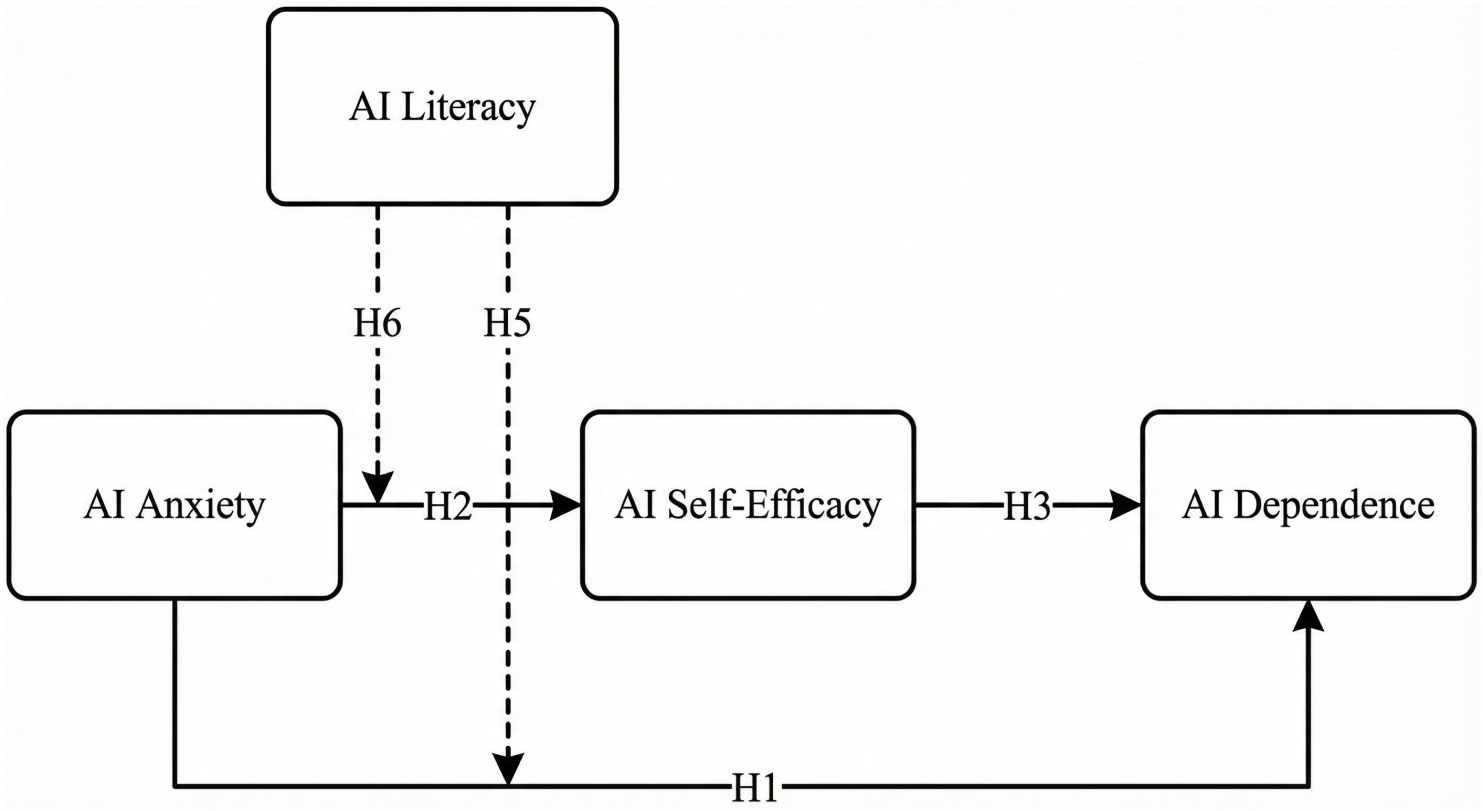
The conceptual model of this study.

Figure 1 presents the conceptual framework developed for the present study based on CBT, COR theory and the aforementioned hypotheses.

## 3 Methodology

This study employed a quantitative cross-sectional survey design to investigate the relationships among AI anxiety, AI self-efficacy, AI literacy, and AI dependence among undergraduates, with particular attention to the mediating effect of AI self-efficacy and the moderated mediation effect involving AI literacy. The research procedure consisted of four major steps. First, eligible participants were recruited through stratified sampling. Second, validated instruments were selected according to the theoretical foundation. Third, an anonymous online questionnaire was administered after ethics approval and informed consent were obtained. Finally, valid responses were screened before measurement validation and hypothesis testing were conducted.

### 3.1 Participants and sampling

The participants were full-time undergraduates from a normal university located in Southwest China. The total undergraduate population was 20,726, and the minimum required sample size was 377 according to the sample size table proposed by Krejcie and Morgan (1970). Considering the study’s focus on AI-related psychological and behavioral constructs, as well as the potential influence of disciplinary background and gender on students’ AI experiences, a stratified sampling strategy was adopted. The sampling strata were defined by major category and gender, and participants were recruited within these strata in proportions that broadly reflected the university’s undergraduate composition. To account for possible non-response and invalid questionnaires, 420 online questionnaires were distributed via Wenjuanxing, a widely used online survey platform in China. Eligible participants were full-time undergraduates at the target university who voluntarily provided informed consent. After data collection, responses were excluded if they were submitted by ineligible respondents, lacked informed consent, contained more than 20% missing data, had completion times of less than 120 s, or showed obvious invalid response patterns such as straight-lining or internally inconsistent answers. Ultimately, 400 valid questionnaires were retained, with an effective response rate of 95.2%. Data collection lasted 2 months between September and November 2025.

As shown in Table 1, the sample consisted of 259 females (64.8%) and 141 males (35.2%). Participants’ ages ranged from 18 to 24 years (*M* = 21.17, SD = 1.51). Students from all academic years were represented, including freshmen (26.8%), sophomores (17.8%), juniors (30.0%), and seniors (25.5%). Regarding major category, natural sciences (40.0%) and social sciences (32.5%) comprised the largest proportions, followed by arts (16.8%) and education (10.8%). With respect to AI usage, 11.0% reported never or rarely using AI tools. Most students reported using AI 1–2 times (45.0%) or 3–4 times (29.0%) per week, and 15.0% indicated more frequent use (≥ 5 times per week).

**TABLE 1:** Descriptive statistics of the samples.

Characteristic	Group	N	Frequency (%)
Gender	Female	259	64.8
	Male	141	35.2
Age	≤ 21	243	60.8
	≥ 22	157	39.2
Academic year	Freshman	107	26.8
	Sophomore	71	17.8
	Junior	120	30.0
	Senior	102	25.5
Major category	Natural Sciences	160	40.0
	Social sciences	130	32.5
	Arts	67	16.8
	Education	43	10.8
Usage of AI (per week)	Never/Rarely	44	11.0
	1–2 times	180	45.0
	3–4 times	116	29.0
	≥ 5 times	60	15.0

The study protocol received approval from the Institutional Review Board (IRB) of the researchers’ university. All participants received an information sheet and provided written informed consent prior to participation. Participation was voluntary and anonymous, and respondents were informed that their data would be used solely for research purposes and that they could withdraw at any time without penalty.

### 3.2 Measures

The four instruments were presented in a fixed, non-randomized sequence that followed the structure of this section, namely AI anxiety, AI self-efficacy, AI literacy, and AI dependence. The construct measured in each section was clearly indicated in the online questionnaire.

#### 3.2.1 AI Anxiety

AI anxiety was measured using the Artificial Intelligence Anxiety Scale (AIAS) developed by Wang and Wang (2022). This 21-item scale was designed to capture individuals’ anxiety regarding AI adoption and development across four dimensions: learning (L), job replacement (JR), sociotechnical blindness (SB), and AI configuration (AIC). Accordingly, the present study operationalized AI anxiety as a general and comprehensive form of AI-related anxiety spanning learning-related, job-related, sociotechnical, and configuration-related concerns, rather than anxiety confined to a single context. Sample items include “Learning to use AI techniques/products makes me anxious” (L), “I am afraid that AI techniques/products will replace someone’s job” (JR), “I am afraid that an AI technique/product may make us dependent” (SB), and “I find humanoid AI techniques/products scary” (AIC). Items were rated on a 7-point Likert scale from 1 (“strongly disagree”) to 7 (“strongly agree”), with higher scores indicating higher levels of AI anxiety. The original study reported excellent internal consistency (Cronbach’s α = 0.96).

In this study, CFA results indicated good construct validity for the AIAS, with χ^2^/df = 2.362, CFI = 0.957, TLI = 0.950, and RMSEA = 0.058. The overall Cronbach’s α was 0.94. The four dimensions also showed satisfactory reliabilities, with Cronbach’s α = 0.94 for L, 0.88 for JR, 0.90 for SB, and 0.87 for AIC.

#### 3.2.2 AI self-efficacy

Undergraduates’ beliefs in their capability to use AI were assessed using the AI Self-Efficacy Scale (AISES) developed by Wang and Chuang (2024). The AISES was specifically developed to capture self-efficacy in relation to distinctive features of AI rather than general computer use. The scale comprises 22 items across four dimensions: assistance (AS), anthropomorphic interaction (ANI), comfort with AI (CF), and technological skills (TS). Example items include “I find that AI technologies/products are helpful for learning” (AS), “I think the interactive process of AI technologies/products is very vivid” (ANI), “When interacting with AI technologies/products, I feel very relaxed” (CF), and “AI technologies/products jargon does not baffle me” (TS). All items were rated on a 7-point Likert scale, with higher scores reflecting stronger AI self-efficacy. In the original validation, the overall Cronbach’s α was 0.96.

In the current study, CFA supported the intended four-factor structure, with χ^2^/df = 2.316, CFI = 0.953, TLI = 0.946, and RMSEA = 0.057, indicating acceptable model fit. The overall internal consistency coefficient was 0.93. Cronbach’s α values for the four dimensions were 0.92 (AS), 0.87 (ANI), 0.91 (CF), and 0.89 (TS), suggesting good reliability for each subscale.

#### 3.2.3 AI literacy

AI literacy was measured using the Artificial Intelligence Literacy Scale (AILS) developed by Wang et al. (2023). This 12-item scale was designed to capture user competence in using AI across four dimensions: awareness (AW), usage (US), evaluation (EV), and ethics (ET). Sample items include “I can distinguish between smart devices and non-smart devices” (AW), “I can use AI applications or products to improve my work efficiency” (US), “I can evaluate the capabilities and limitations of an AI application or product after using it for a while” (EV), and “I always comply with ethical principles when using AI applications or products” (ET). Items were rated on a 7-point Likert scale from 1 (“strongly disagree”) to 7 (“strongly agree”), with higher scores indicating higher levels of AI literacy. The original study reported good internal consistency (Cronbach’s α = 0.83).

In this study, CFA results indicated good construct validity for the AILS, with χ^2^/df = 2.961, CFI = 0.959, TLI = 0.943, and RMSEA = 0.070. The overall Cronbach’s α was 0.90. The four dimensions also showed satisfactory reliabilities, with Cronbach’s α = 0.83 for AW, 0.77 for US, 0.85 for EV, and 0.80 for ET.

#### 3.2.4 AI dependence

AI dependence was assessed using the AI Dependence Scale (AIDep-22) developed by Wu et al. (2026). This 22-item scale was designed to capture students’ overreliance on AI across four dimensions: emotional dependence (ED), functional dependence (FD), cognitive dependence (CD), and loss of control (LC). Example items include “I feel uneasy or insecure without the help of AI” (ED), “I delegate almost all my academic tasks to AI because it performs them faster and better than I do” (FD), “I often ask AI for answers directly without thinking for myself first” (CD), and “I have tried to reduce my use of AI, but I have never succeeded for long” (LC). All items were rated on a 5-point Likert scale from 1 (“strongly disagree”) to 5 (“strongly agree”), with higher scores reflecting higher levels of AI dependence. In the original validation, the overall Cronbach’s α was 0.87.

In the current study, CFA supported the intended four-factor structure, with χ^2^/df = 2.092, CFI = 0.955, TLI = 0.949, and RMSEA = 0.052, indicating acceptable model fit. The overall internal consistency coefficient was 0.93. Cronbach’s α values for the four dimensions were 0.88 (ED), 0.87 (FD), 0.88 (CD), and 0.89 (LC), suggesting good reliability for each subscale.

#### 3.2.5 Control variables

Previous studies indicate that demographic and usage-related characteristics may influence students’ AI-related experiences (Sunmboye et al., 2025). In this study, gender, age, academic year, major category, and weekly AI use frequency were included as control variables in the model.

### 3.3 Data analysis

The data were analyzed using SPSS 27.0, Amos 27.0 and the PROCESS macro (Hayes, 2017). The analysis followed a systematic four-step procedure.

First, CFA and reliability analysis were conducted to verify the validity and internal consistency of the AIAS, AISES, AILS, and AIDep-22 instruments within the current sample. Second, Harman’s single-factor test was employed to assess the potential for common method bias (CMB), given that all data were self-reported. Third, descriptive statistics and Pearson correlation coefficients were calculated to provide an overview of the central tendencies and bivariate relationships among AI anxiety, AI self-efficacy, AI literacy, and AI dependence.

Finally, hypothesis testing was conducted using the PROCESS macro. Model 4 was utilized to test the direct effect of AI anxiety on AI dependence and the mediating role of AI self-efficacy (Hypotheses 1–4). Subsequently, a moderated mediation analysis was performed (Model 8) to examine whether AI literacy moderates the relationship between AI anxiety and AI dependence, and the relationship between AI anxiety and AI self-efficacy (Hypotheses 5–6). The bootstrap method with 5,000 resamples was used to generate 95% confidence intervals (CI) for the effects. If the CI did not include zero, the effect was considered statistically significant.

Before hypothesis testing, regression assumptions for the PROCESS models were examined. The main variables showed acceptable normality, with skewness ranging from –0.139 to 0.196 and kurtosis ranging from –0.355 to 0.109. The standardized residuals of the PROCESS regression equations also showed acceptable normality, with skewness ranging from –0.108 to 0.031 and kurtosis ranging from –0.221 to 0.101. Standardized residual plots indicated no obvious curvilinear or funnel-shaped pattern, and Breusch-Pagan tests were non-significant across the regression equations, with *p*-values ranging from 0.514 to 0.940. Multicollinearity was not a concern because the variance inflation factor values ranged from 1.01 to 1.78. These results indicated that the assumptions of normality, linearity, homoscedasticity, and multicollinearity were adequately met before the mediation and moderated mediation analyses were conducted. Detailed SPSS diagnostic outputs for the PROCESS models are provided in the Supplementary material.

## 4 Results

### 4.1 Common method bias test

Procedural remedies were applied during survey design, including anonymous participation and the inclusion of reverse-scored items. Statistically, Harman’s single-factor test was conducted on all measurement items (77 items in total) using an unrotated factor extraction. The analysis extracted 15 factors with eigenvalues greater than 1.0, and the first factor accounted for 27.17% of the total variance, which is well below the commonly accepted 40% criterion. Therefore, the results indicate that the present study does not exhibit a significant risk of common method bias.

### 4.2 Descriptive statistics and correlation analysis

Table 2 reports the descriptive statistics and Pearson correlations for the main variables. Students reported moderate levels of AI anxiety (*M* = 3.62, SD = 0.98) and AI dependence (*M* = 3.12, SD = 0.82), alongside relatively high AI self-efficacy (*M* = 4.42, SD = 0.91) and AI literacy (*M* = 4.82, SD = 0.84). Correlation results showed that AI anxiety was significantly and positively related to AI dependence (*r* = 0.528, *p* < 0.001), while it was negatively related to AI self-efficacy (*r* = –0.484, p < 0.001) and AI literacy (*r* = –0.431, *p* < 0.001). In addition, AI self-efficacy and AI literacy were positively correlated (*r* = 0.516, *p* < 0.001), and both were negatively associated with AI dependence (*r* = –0.492 and –0.530, respectively, *p* < 0.001). Cronbach’s α coefficients indicated good internal consistency for all scales (α = 0.90—0.94).

**TABLE 2:** Descriptive statistics and correlations among the variables.

Variable	M	SD	α	1	2	3	4
1. AI anxiety	3.62	0.98	0.94	1	1	1	1
2. AI self-efficacy	4.42	0.91	0.93	–0.484***			
3. AI literacy	4.82	0.84	0.90	–0.431***	0.516***		
4. AI dependence	3.12	0.82	0.93	0.528***	–0.492***	–0.530***	

*N* = 400. M, mean; SD, standard deviation; α, Cronbach’s alpha.

***p < 0.001.

### 4.3 The mediating role of AI self-efficacy

Model 4 of the PROCESS macro in SPSS was used to test the mediation model. In this model, AI anxiety was the independent variable, AI dependence was the dependent variable, and AI self-efficacy was the mediating variable. Gender, age, academic year, major, and weekly AI use were included as control variables.

The control variables did not show significant effects across the mediation model pathways. In the total effect model predicting AI dependence, gender, age, academic year, major, and weekly AI use were all non-significant, with p values ranging from 0.186 to 0.940. In the mediator model predicting AI self-efficacy, the same control variables were also non-significant, with *p*-values ranging from 0.235 to 0.869. In the full outcome model including both AI anxiety and AI self-efficacy, none of the control variables significantly predicted AI dependence, with *p*-values ranging from 0.181 to 0.819. These results indicate that the observed mediation pattern was not primarily attributable to demographic characteristics or weekly AI use frequency.

As shown in Table 3, AI anxiety significantly and positively predicted AI dependence in the total effect model (β = 0.53, *p* < 0.001). In the mediation model, AI anxiety significantly and negatively predicted AI self-efficacy (β = –0.48, *p* < 0.001). When both AI anxiety and AI self-efficacy were entered into the full model, AI self-efficacy significantly and negatively predicted AI dependence (β = –0.31, *p* < 0.001), while the positive effect of AI anxiety on AI dependence remained significant (β = 0.38, *p* < 0.001). These results support H1–H3.

**TABLE 3:** Test of the mediating effect of AI self-efficacy (Model 4).

Outcome variable	*R*	*R* ^2^	*F*	Predictor	β	SE	*t*	*p*
AI dependence	0.53	0.28	25.96***	Gender	–0.01	0.07	–0.23	0.820
				Age	–0.05	0.03	–0.82	0.412
				Academic year	0.07	0.04	1.33	0.186
				Major	0.05	0.04	1.15	0.252
				AI use	–0.00	0.04	–0.08	0.940
				AIA	0.53***	0.04	12.30***	< 0.001
AI self-efficacy	0.49	0.24	20.75***	Gender	–0.03	0.09	–0.69	0.490
				Age	0.02	0.04	0.39	0.697
				Academic year	–0.01	0.05	–0.17	0.869
				Major	–0.05	0.04	–1.19	0.235
				AI use	0.04	0.04	0.92	0.356
				AIA	–0.48***	0.04	–10.94***	< 0.001
AI dependence	0.60	0.36	30.90***	Gender	–0.02	0.07	–0.47	0.639
				Age	–0.04	0.03	–0.73	0.463
				Academic year	0.07	0.04	1.34	0.181
				Major	0.03	0.03	0.81	0.418
				AI use	0.01	0.04	0.23	0.819
				AIA	0.38***	0.04	8.14***	< 0.001
				AISE	–0.31***	0.04	–6.61***	< 0.001

*N* = 400. AIA, AI Anxiety; AISE, AI Self-efficacy; β represents standardized coefficients; SE represents standard errors.

***p < 0.001.

**FIGURE 2:**
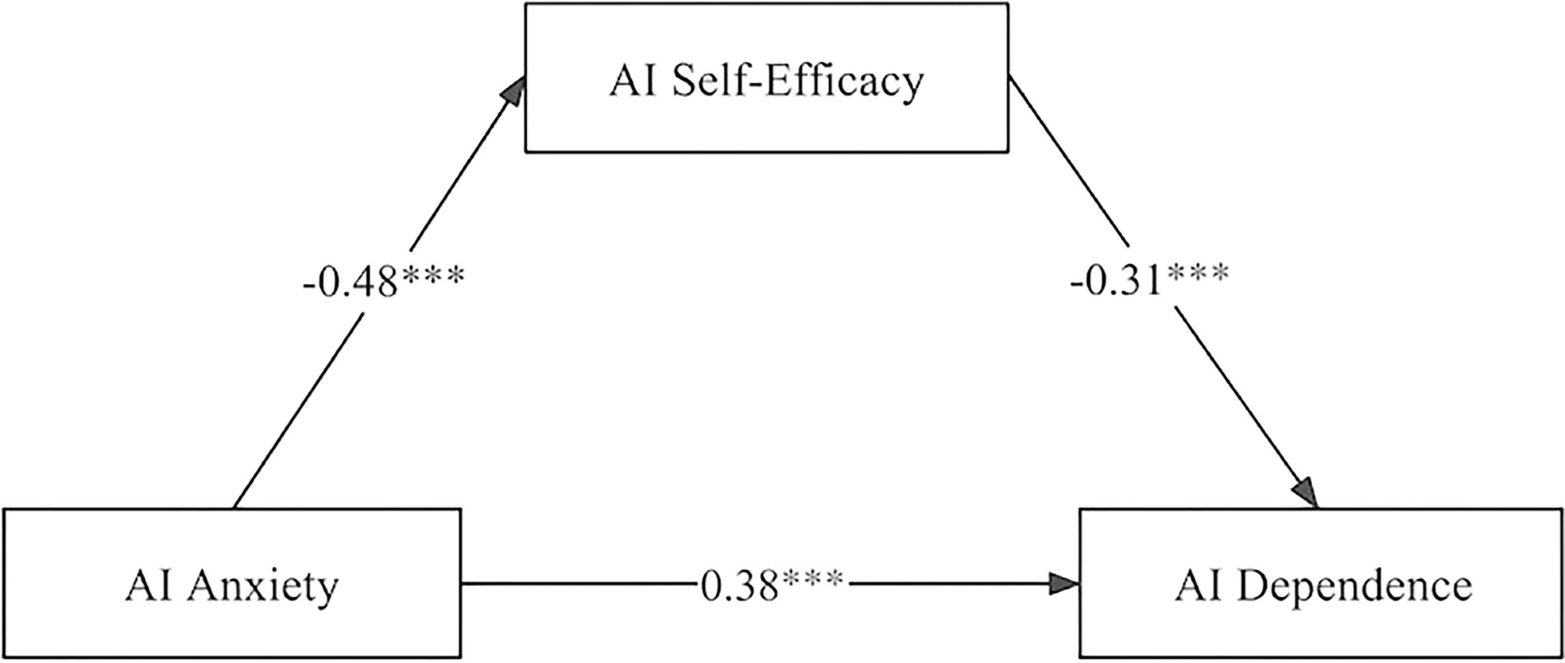
Path diagram of the mediating role of AI self-efficacy.

The mediating effect was further tested using bias-corrected bootstrap (5,000 samples). As shown in Table 4, the indirect effect of AI anxiety on AI dependence through AI self-efficacy was significant, with the 95% CI excluding 0. Thus, H4 was supported. The structural relationships and specific path coefficients among these variables are visually illustrated in Figure 2.

**TABLE 4:** Effects of AI anxiety on AI dependence via AI self-efficacy (Model 4).

Path	Effect	SE	95% CI (Bias-corrected)	Ratio of total effect
Total effect	0.44	0.04	[0.37, 0.51]	–
Direct effect	0.32	0.04	[0.24, 0.39]	71.84%
Indirect effect	0.12	0.02	[0.08, 0.17]	28.16%

Effects (b), standard errors (SE), and confidence intervals (CI) are unstandardized. Indirect effect estimates are based on 5,000 bias-corrected bootstrap samples.

### 4.4 The moderating role of AI literacy

Model 8 of the PROCESS macro program was used to test the moderating effect of AI literacy. In this analysis, AI anxiety was the independent variable, AI dependence was the dependent variable, AI self-efficacy was the mediating variable, and AI literacy served as the moderator, and other demographic variables were controlled.

Table 5 shows that the interaction between AI anxiety and AI literacy did not significantly predict AI dependence (*b* = 0.04, *p* > 0.05). However, the interaction term significantly predicted AI self-efficacy (*b* = –0.11, *p* < 0.01), indicating that AI literacy moderates the relationship between AI anxiety and AI self-efficacy. Therefore, H6 was supported, whereas H5 was not supported.

**TABLE 5:** Test of the moderating effect of AI literacy (Model 8).

Outcome variable	*R*	*R* ^2^	*F*	Predictor	*B*	SE	*t*	*p*
AI self-efficacy	0.61	0.37	28.31***	Gender	–0.08	0.08	–1.03	0.303
				Age	0.02	0.03	0.51	0.608
				Academic year	–0.00	0.04	–0.06	0.949
				Major	–0.01	0.04	–0.23	0.818
				AI use	0.03	0.04	0.76	0.447
				AIA	–0.30***	0.04	–7.33***	<0.001
				AIL	0.41***	0.04	8.42***	< 0.001
				AIA × AIL	–0.11**	0.04	–2.72**	0.007
AI dependence	0.65	0.42	31.12***	Gender	–0.01	0.07	–0.10	0.918
				Age	–0.02	0.03	–0.80	0.426
				Academic year	0.05	0.04	1.32	0.187
				Major	0.00	0.03	0.12	0.908
				AI use	0.01	0.03	.20	0.838
				AISE	–0.16***	0.04	–3.74***	<0.001
				AIA	0.26***	0.04	6.82***	<0 001
				AIL	–0.29***	0.04	–6.43***	<0.001
				AIA × AIL	0.04	0.03	1.04	0.300

*N* = 400. AIA, AI Anxiety; AISE, AI Self-efficacy; AIL, AI Literacy; b represents unstandardized coefficients; SE represents standard errors.

**p < 0.01, ***p < 0.001.

**FIGURE 3:**
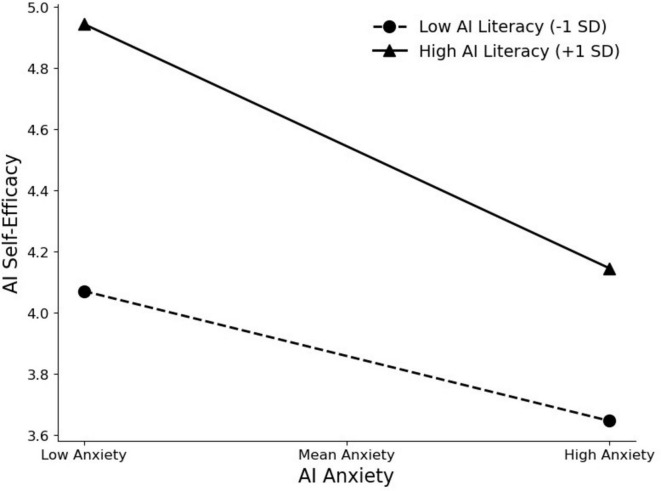
Simple slope of the moderating effect of AI literacy.

Further simple slope analysis was conducted as shown in Figure 3, illustrating that the negative association between AI anxiety and AI self-efficacy intensified as AI literacy levels increased. This indicates that students with high AI literacy are more sensitive to the adverse effects of AI anxiety on their self-efficacy compared to their counterparts with low AI literacy. Additionally, as shown in Table 6, the index of moderated mediation was significant (Index = 0.018, 95% CI [0.005, 0.036]), indicating that AI literacy conditionally alters the indirect effect of AI anxiety on AI dependence through AI self-efficacy.

**TABLE 6:** Effects of AI anxiety on AI dependence via AI self-efficacy by AI literacy (Model 8).

AI literacy level	Effect	BootSE	BootLLCI	BootULCI
Low (–1 SD)	0.035	0.013	0.014	0.064
High (+1 SD)	0.065	0.021	0.030	0.111
Index of moderated mediation	0.018	0.008	0.005	0.036

Effects are based on 5,000 bootstrap samples. LLCI, Lower Limit Confidence Interval; ULCI, Upper Limit Confidence Interval.

The effects of the control variables were also examined in the moderated mediation model. In the equation predicting AI self-efficacy, gender, age, academic year, major, and weekly AI use were not significant predictors, with *p*-values ranging from 0.303 to 0.949. In the equation predicting AI dependence, these control variables again showed no significant effects, with *p*-values ranging from 0.187 to 0.918. Therefore, the significant interaction between AI anxiety and AI literacy on AI self-efficacy, as well as the conditional indirect effect, remained interpretable after accounting for demographic and usage-related characteristics.

## 5 Discussion

This study was designed to elucidate the psychological mechanisms linking AI anxiety to AI dependence by grounding the analysis in the frameworks of CBT and COR theory. By testing a moderated mediation model, the findings not only corroborate the central mediating role of AI self-efficacy in explaining how compromised competence beliefs translate anxiety into dependence but, more critically, clarify the boundary conditions established by AI literacy. Specifically, the analysis reveals that AI literacy functions as a pivotal personal resource that significantly conditions the relationship between AI anxiety and AI self-efficacy. The results provide nuanced theoretical insights that move beyond simple correlational descriptions and demonstrate how cognitive resources interact with emotional states to shape human-AI interaction patterns.

First, the results indicated that AI anxiety was significantly and positively related to students’ AI dependence. Students with higher AI anxiety reported greater dependence, and the correlation was moderate in magnitude, which suggests that anxiety is not a trivial correlate but a substantive psychological condition associated with overreliance. This finding fits the core idea in CBT that affective distress can shift individuals toward short term coping patterns, which reduce perceived uncertainty and effort, especially when tasks are cognitively demanding and outcomes matter for self-evaluation (Stentz and Cougle, 2022). In higher education, generative AI is often treated as a high impact learning tool, but it is also experienced as an opaque system in which outputs can be unpredictable and academic consequences are ambiguous (Essien et al., 2024). Under such conditions, AI anxiety can function as an affective signal that narrows attention toward immediate relief and completion, which increases the likelihood of delegating thinking or decision making to AI (Yu et al., 2024). This interpretation is consistent with recent research on AI dependence that emphasizes its cognitive and functional features that distinguish dependence from high frequency usage. Specifically, AI dependence is linked to patterns such as delegating academic tasks and reducing independent cognitive effort, rather than simply spending more time with AI (Hou et al., 2025). This conceptualization helps clarify why anxiety is a plausible driver of dependence in students who may be trying to reduce risk and uncertainty in learning tasks.

Second, the results indicated that AI self-efficacy mediated the relationship between AI anxiety and AI dependence. This implies that AI anxiety can indirectly increase dependence by weakening students’ confidence in their ability to use AI in a controlled and effective manner. On the one hand, AI self-efficacy was significantly and negatively associated with AI anxiety. This pattern is consistent with the broader self-efficacy literature in technology contexts, where negative arousal tends to be interpreted as a cue of insufficient capability, which reduces perceived competence and control (Truţa et al., 2023). When students feel tense, confused, or worried about AI, they may infer that they cannot manage prompts, evaluate outputs, or integrate AI appropriately into their academic work, which erodes their efficacy beliefs (Chen et al., 2024). On the other hand, AI self-efficacy was significantly and negatively associated with AI dependence, which suggests that confidence beliefs are protective against overreliance. Students who believe they can use AI competently are more likely to keep themselves cognitively engaged, treat AI as assistance rather than substitution, and maintain verification habits that prevent blind reliance (Chong et al., 2022). Importantly, the mediation effect was partial, which indicates that efficacy based coping explains only part of how AI anxiety translates into dependence. The remaining portion may reflect additional appraisal linked routes that were not modeled in the current study, such as perceived academic pressure, fear of evaluation, or avoidance oriented coping (Gu and Mao, 2023). This partial mediation pattern is theoretically meaningful as it supports the CBT sequence specified in the literature review while also indicating that dependence can emerge from multiple pathways in the broader academic environment.

Third, the AI literacy related findings reveal a nuanced boundary condition that refines the COR theory based argument in the literature review. AI literacy was strongly related to both AI self-efficacy and AI dependence at the bivariate level, and it remained a significant predictor in the moderated mediation model. However, the interaction between AI anxiety and AI literacy did not significantly predict AI dependence, which indicates that literacy does not weaken the direct translation of anxiety into dependence in this sample. At the same time, AI literacy significantly moderated the AI anxiety to AI self-efficacy path. This pattern suggests that AI literacy operates primarily at the cognitive appraisal stage, shaping how anxiety affects competence beliefs rather than directly blocking anxiety driven reliance behavior. This is consistent with COR logic as resources often influence stress processes by altering perceived controllability and coping capacity, which then affects downstream behavior (Demerouti, 2025). In practical terms, AI literacy may help students interpret AI outputs more critically and understand limitations, which protects efficacy when anxiety arises (Liu, 2026). However, AI anxiety can still motivate dependence behaviors directly, particularly under deadlines or performance pressure, because affective urgency can override knowledge based self-regulation instantaneously (Quinn and Shields, 2023).

Finally, the conditional effects and the significant moderated mediation index indicate that the indirect effect of AI anxiety on AI dependence through self-efficacy varies with AI literacy. In this study, the indirect effect was larger at higher AI literacy, which differs from a simple buffering expectation. A plausible explanation is that among students with high AI literate, efficacy beliefs become more central in regulating how AI is used (Kong et al., 2025). When students know more about AI, they may also hold higher standards for what competent use entails, including prompt planning, verification, and ethical compliance (Zhou et al., 2025). Under AI anxiety, these students may be more likely to interpret their distress as evidence that they are falling short of those standards, which can reduce self-efficacy more strongly and amplify the indirect pathway (Chen et al., 2025). This interpretation does not contradict COR. It indicates that resources can shape the meaning of anxiety and the diagnostic value of affective cues, not only the magnitude of stress exposure (Tuan, 2022). Future studies should test this explanation directly by separating literacy dimensions, measuring perceived controllability, and examining whether self-evaluation standards mediate the literacy related differences in the anxiety to efficacy slope.

Taken together, these findings extend the literature by empirically linking AI anxiety and AI dependence through a competence based mechanism, and by specifying where AI literacy conditions the process. The results suggest that undergraduates’ AI dependence is not only a matter of convenience or habit. It is partly embedded in anxiety-driven coping and efficacy related self-regulation, which are increasingly salient as generative AI becomes a routine component of higher education.

## 6 Implications

Theoretically, this study advances research on undergraduates’ AI adaptation by clarifying AI dependence as an anxiety sensitive outcome that is partly shaped by competence beliefs. By grounding the model in CBT and COR theory, it emphasizes that AI dependence is not solely determined by availability or perceived utility. It is also shaped by how students appraise AI related threats and by the cognitive resources they can mobilize to cope with those threats. The results provide direct evidence that AI self-efficacy transmits part of the effect of AI anxiety to AI dependence, thereby operationalizing the emotion to cognition to behavior sequence that was proposed in the literature review (Chen et al., 2024; Wang et al., 2020; Zhang et al., 2024). The findings also refine the role of AI literacy by showing that it conditions the AI anxiety to AI self-efficacy pathway rather than the direct AI anxiety to AI dependence link. This distinction extends the boundary condition argument by indicating that AI literacy primarily influences appraisal and competence belief dynamics, which then shape reliance behaviors.

Practically, the findings suggest that universities should address AI dependence through an integrated strategy that targets AI anxiety, AI self-efficacy, and AI literacy simultaneously. As AI anxiety is moderately associated with AI dependence, student support services and academic units should treat AI anxiety as a real adaptation pressure rather than as a marginal attitude. Universities can reduce threat appraisal by clarifying acceptable academic use, providing stable guidance on integrity and evaluation standards, and designing learning environments where experimentation with AI is not automatically equated with misconduct or incompetence. At the same time, the mediation findings indicate that strengthening AI self-efficacy is a concrete pathway for reducing dependence. Training should prioritize controllable skills such as prompt formulation, verification routines, error detection, and reflective integration of AI into problem-solving rather than simple tool demonstrations. These competence building experiences can help students experience mastery and reduce the tendency to outsource thinking when anxious.

The moderation findings also imply that AI literacy programs should be designed not only to increase knowledge but also to protect efficacy under stress. AI literacy instruction that emphasizes limitations, uncertainty, and ethical risks may unintentionally raise standards and self-evaluative pressure if it is delivered without adequate scaffolding for skill mastery (Zhu et al., 2025). Therefore, AI literacy education should be paired with efficacy building practice and feedback so that students learn to manage limitations without losing confidence. In addition, because the direct AI anxiety to AI dependence link was not moderated by AI literacy, universities should not assume that knowledge alone prevents overreliance. Emotional and motivational support, time management design, and assessment formats that reward process and reasoning are also needed to reduce anxiety-driven delegation behaviors.

## 7 Conclusion

This study addresses the relationship between AI anxiety and AI dependence among undergraduates. Grounded in CBT and COR theory, the research constructed and validated a moderated mediation model to elucidate how affective strain translates into behavioral reliance. The findings confirm that AI anxiety is a significant positive predictor of AI dependence, suggesting that students’ overreliance on AI tools is often a maladaptive coping response to technological apprehension rather than merely a pursuit of utility. Crucially, AI self-efficacy was identified as a partial mediator, revealing that anxiety exacerbates dependence by eroding students’ confidence in their own capabilities. Furthermore, AI literacy emerged as a pivotal boundary condition that moderates the anxiety-efficacy pathway. Contrary to a simple buffering hypothesis, higher AI literacy was found to sensitize students to the competency gaps induced by anxiety, thereby intensifying the indirect effect on dependence. These results advance theory by integrating affective strain, competence beliefs, and capability resources into a single explanatory framework for undergraduates’ AI dependence. Practically, the findings provide an evidence-based basis for universities to design more effective student support and AI-integration policies.

Several limitations warrant consideration for future research. First, the cross-sectional design restricts causal inferences regarding the temporal sequence from anxiety to dependence; longitudinal studies are needed to track these dynamics across academic semesters. Second, the sample was drawn from a single normal university, which limits generalizability; future work should replicate the model across diverse institution types and cultural contexts. Third, reliance on self-reported measures may introduce bias; integrating objective behavioral indicators, such as platform logs or performance-based tasks, would strengthen validity. Fourth, the four questionnaires were presented in a fixed order rather than a randomized or counterbalanced sequence. Although participants were clearly informed about the construct measured in each section, this procedure may have introduced order, priming, or fatigue effects. Future studies should randomize or counterbalance questionnaire order to reduce potential procedural bias. Finally, the complex moderating role of AI literacy suggests the need for further decomposition of literacy dimensions and the inclusion of additional contextual variables, such as academic workload and integrity standards, to fully capture the ecological drivers of AI dependence. Despite these limitations, this study provides robust, theory-grounded evidence that AI dependence is an anxiety-driven phenomenon shaped by the interplay of AI self-efficacy and AI literacy, offering a refined roadmap for supporting undergraduates’ adaptive engagement with AI.

## Data Availability

The raw data supporting the conclusions of this article will be made available by the authors, without undue reservation.
