# Supplementary material for: AI anxiety and AI dependence among undergraduates: a moderated mediation model of AI self-efficacy and AI literacy
Source: Front Psychol. 2026 Jul 13;17:1884382. doi: 10.3389/fpsyg.2026.1884382 (PMC13416768; doi:10.3389/fpsyg.2026.1884382)

Regression Assumptions for the PROCESS Models

Before hypothesis testing, regression assumptions for the PROCESS models were examined. The main variables showed acceptable normality, with skewness ranging from -0.139 to 0.196 and kurtosis ranging from -0.355 to 0.109. The standardized residuals of the PROCESS regression equations also showed acceptable normality, with skewness ranging from -0.108 to 0.031 and kurtosis ranging from -0.221 to 0.101. Standardized residual plots indicated no obvious curvilinear or funnel-shaped pattern, and Breusch-Pagan tests were non-significant across the regression equations, with p values ranging from .514 to .940. Multicollinearity was not a concern because the variance inflation factor values ranged from 1.01 to 1.78. These results indicated that the assumptions of normality, linearity, homoscedasticity, and multicollinearity were adequately met before the mediation and moderated mediation analyses were conducted. **All test output from SPSS as shown in the following tables and figures.**

Table S1. Descriptive statistics and normality of main variables

Variable	N	M	SD	Skewness	SE	Kurtosis	SE
AI anxiety	400	3.62	0.98	0.196	0.122	-0.279	0.243
AI self-efficacy	400	4.42	0.91	0.089	0.122	-0.355	0.243
AI literacy	400	4.82	0.84	-0.139	0.122	0.109	0.243
AI dependence	400	3.12	0.82	-0.085	0.122	-0.277	0.243

Note. Values are consistent with SPSS descriptive output. Skewness and kurtosis values within ±2 indicate acceptable normality for regression-based analysis.

Table S2. Regression assumption diagnostics for PROCESS models

PROCESS Equation	R^2	Durbin-Watson	Residual Skewness	Residual Kurtosis	Breusch-Pagan p	Min Tolerance	Max VIF	Interpretation
Model 4, mediator equation, AISE	.241	1.850	-0.108	0.101	.918	.567	1.764	Assumptions met
Model 4, outcome equation, AID	.356	1.921	-0.025	-0.221	.514	.567	1.764	Assumptions met
Model 8, mediator equation, AISE	.367	1.912	-0.101	-0.083	.821	.563	1.776	Assumptions met
Model 8, outcome equation, AID	.418	1.823	0.031	-0.169	.940	.563	1.778	Assumptions met

Note. AISE = AI self-efficacy. AID = AI dependence. Breusch-Pagan p values above .05 indicate no significant heteroscedasticity. VIF values below 5 indicate no multicollinearity concern.

Figure S1. Normal P-P Plot for Model 4 Mediator Equation

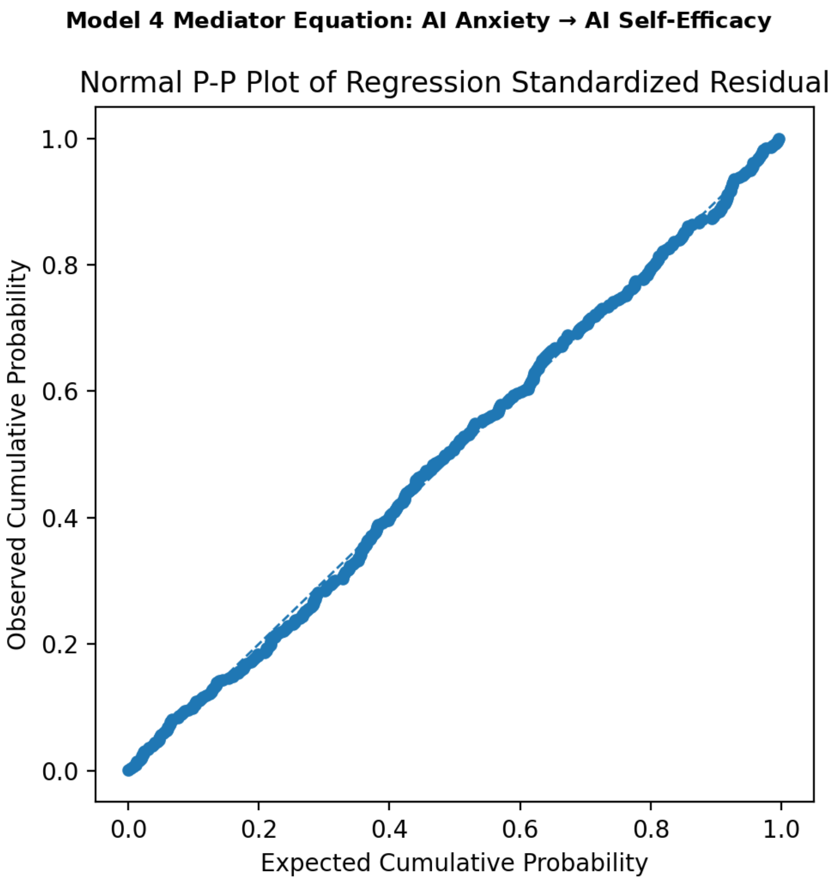

Figure S2. Scatterplot of Standardized Residuals Against Standardized Predicted Values for Model 4 Mediator Equation

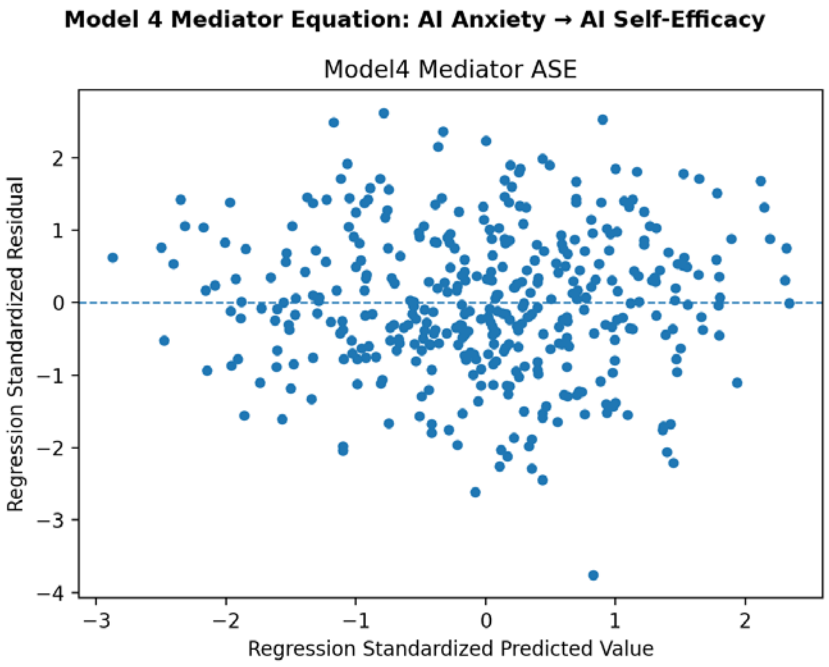

Figure S3. Normal P-P Plot for Model 4 Outcome Equation

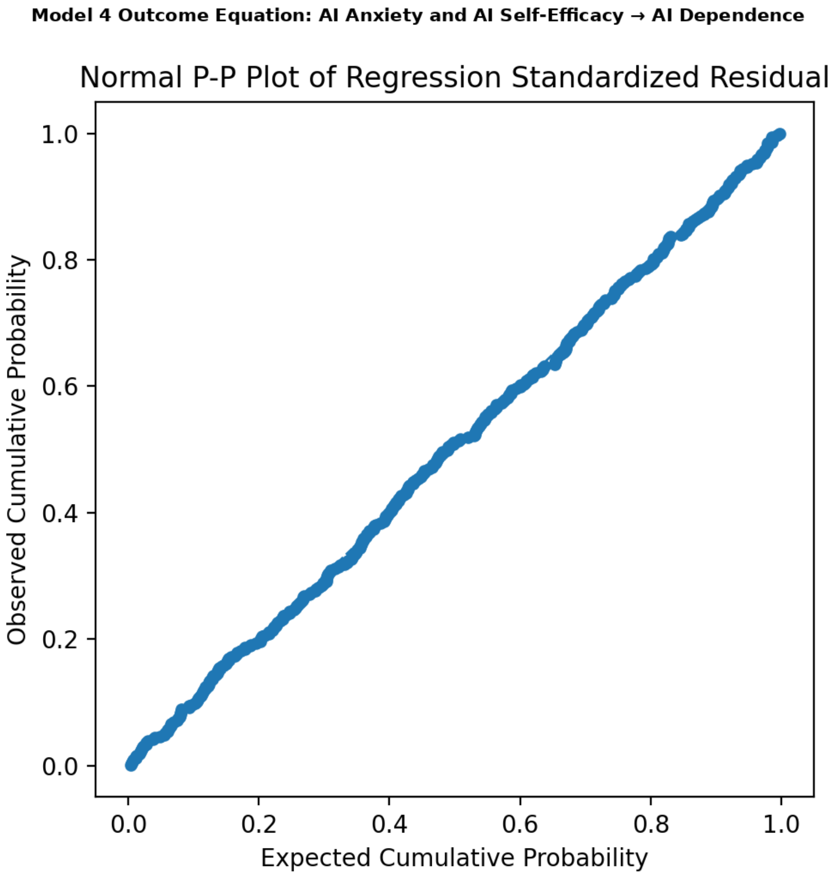

Figure S4. Scatterplot of Standardized Residuals Against Standardized Predicted Values for Model 4 Outcome Equation

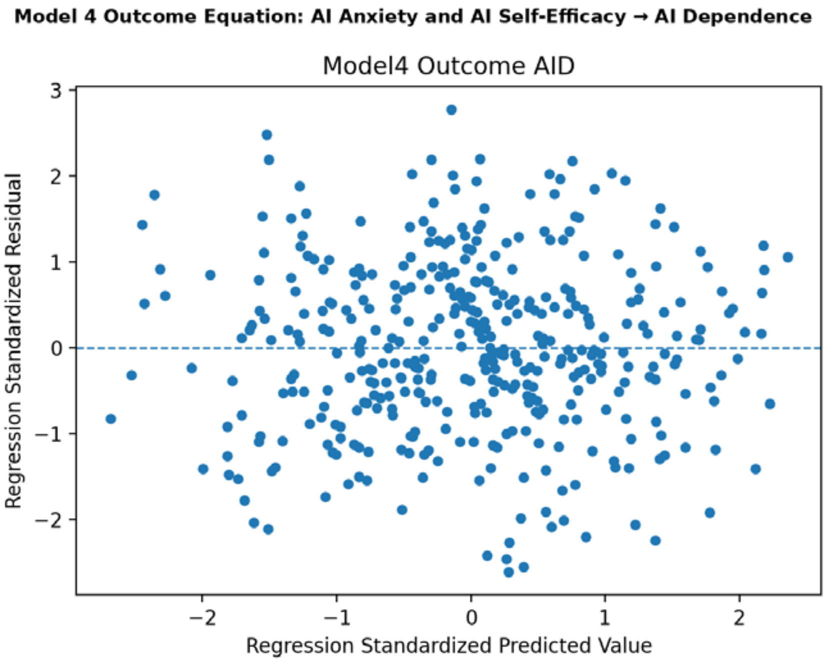

Figure S5. Normal P-P Plot for Model 8 Mediator Equation

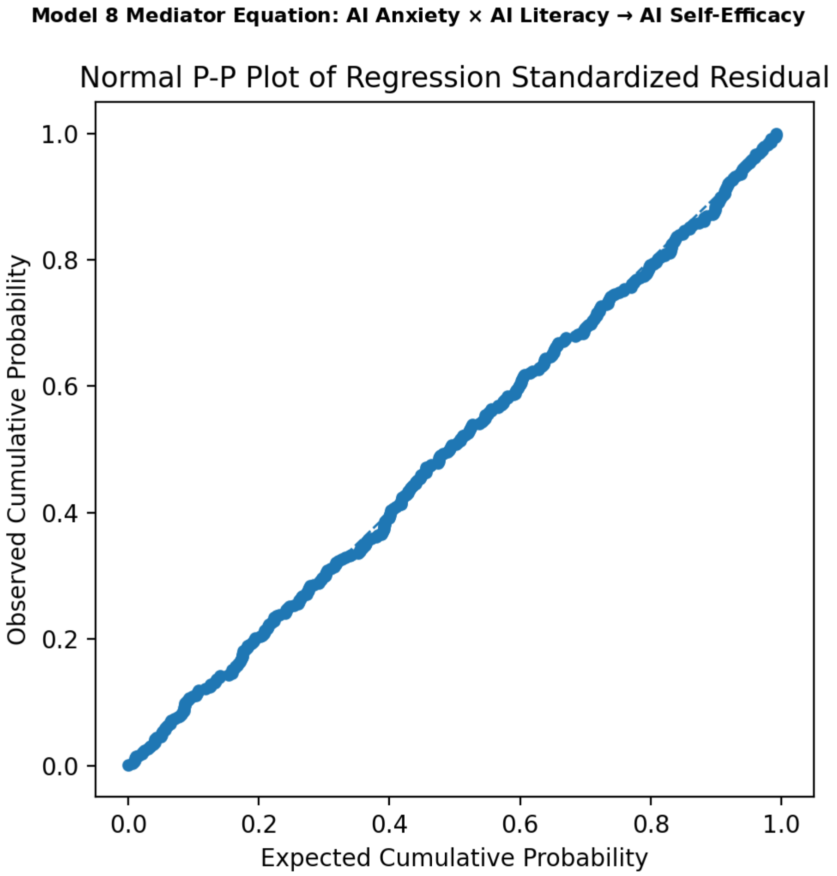

Figure S6. Scatterplot of Standardized Residuals Against Standardized Predicted Values for Model 8 Mediator Equation

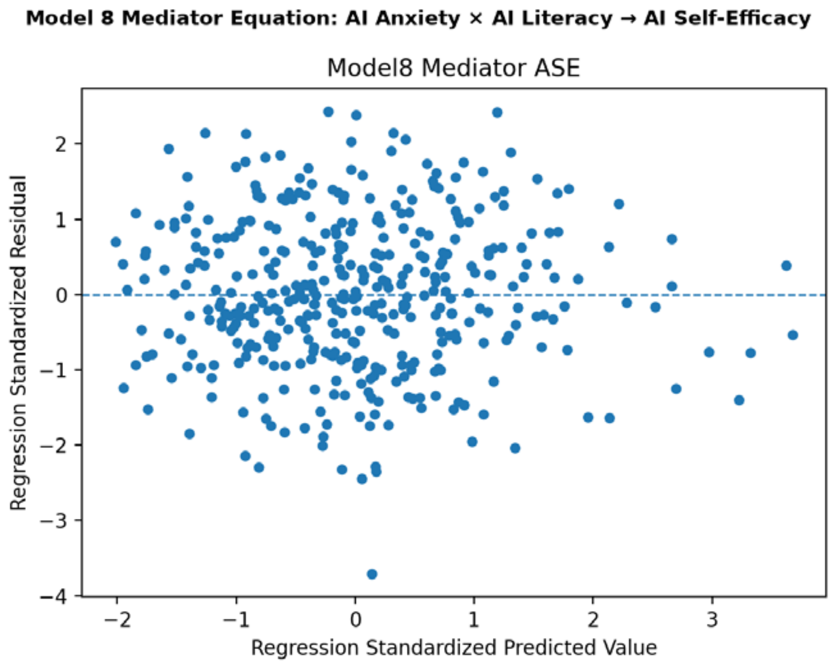

Figure S7. Normal P-P Plot for Model 8 Outcome Equation

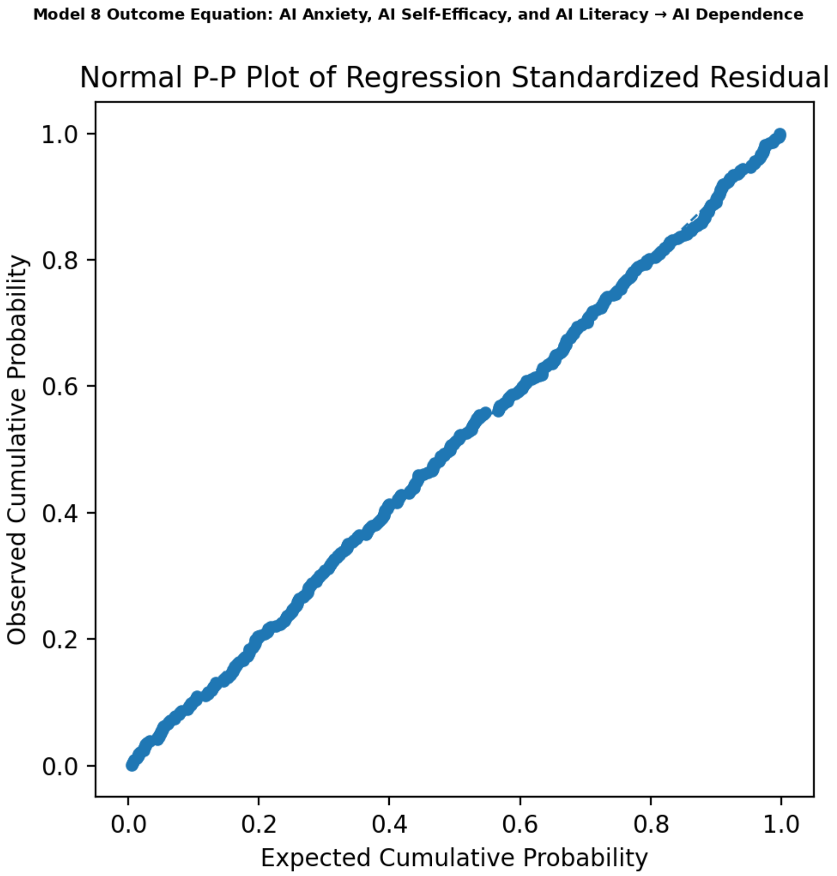

Figure S8. Scatterplot of Standardized Residuals Against Standardized Predicted Values for Model 8 Outcome Equation

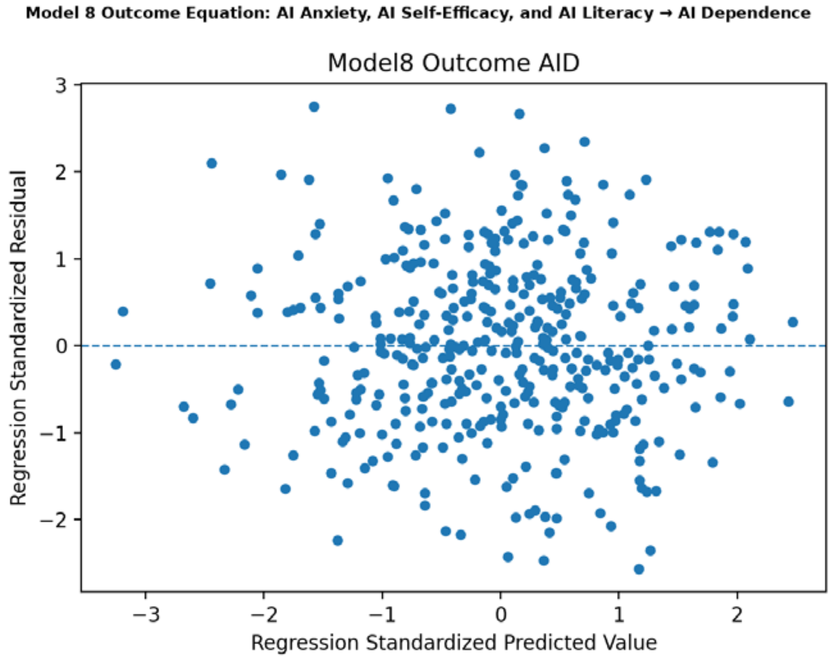

Supplement: Supplementary file 1 [file Data_Sheet_1.pdf]
